# Diminished anticipatory-consummatory pleasure interplay in high schizotypal traits and subthreshold depression: potential risk for schizophrenia and depression

**DOI:** 10.1038/s41537-026-00746-x

**Published:** 2026-04-04

**Authors:** Lei Shen, Chi Xie, Chang Liu, Yan-yu Wang, Yi Wang, Chao Yan, Raymond C. K. Chan

**Affiliations:** 1 Key Laboratory of Brain Functional Genomics (MOE&STCSM), Affiliated Mental Health Center (ECNU), School of Psychology and Cognitive Science, East China Normal University, Shanghai, China; 2 Shanghai Changning Mental Health Center, Shanghai, China; 3 Mental Health Centre of Weifang city, Weifang, Shandong China; 4 School of Psychology, Shandong Second Medical University, Weifang, Shandong China; 5 Neuropsychology and Applied Cognitive Neuroscience Laboratory; CAS Key Laboratory of Mental Health, Institute of Psychology, Chinese Academy of Sciences, Beijing, China; 6 Department of Psychology, University of Chinese Academy of Sciences, Beijing, China

**Keywords:** Schizophrenia, Human behaviour

## Abstract

Anhedonia is a multidimensional construct encompassing anticipatory and consummatory pleasure, and it manifests across a range of psychiatric disorders including schizophrenia (SCZ) and major depressive disorder (MDD). However, the shared and distinct mechanisms that underlie anhedonia between SCZ and MDD remain unclear. This study explored the characteristics and interplay between anticipatory and consummatory affective responses in SCZ and MDD patients as well as their subclinical counterparts. A total of 821 participants completed the Monetary Incentive Delay task. Network analysis was conducted on the full sample. For group comparisons, 59 non-clinical participants were excluded due to missing classification data or overlapping subclinical criteria, yielding 762 participants (115 SCZ, 60 MDD, 83 schizotypal traits (ST), 110 subthreshold depression (SD), and 394 healthy controls (HC)). Partial correlation networks were constructed using anticipatory and consummatory affective responses, calculated by averaging the valence subjective rating scores as nodes. Separate network comparisons were conducted to examine group differences within the subgroups. The results showed that consummatory affective responses under neutral condition was the central in the network. The global strength of network was lower in the both ST and SD participants compared to SCZ and MDD patients, respectively. Both SCZ patients and ST individuals showed stronger connections between anticipatory and consummatory pleasure compared to MDD and SD individuals. Our findings suggested that connections of multidimensional components of anhedonia played crucial role in the transdiagnostic network and the inter-relationship between anticipatory and consummatory pleasure might underlie the distinct mechanisms of anhedonia in SCZ and MDD.

## Introduction

Anhedonia, traditionally defined as a diminished experience of pleasure, is increasingly recognized as a multidimensional construct encompassing deficits not only in the hedonic response itself but also in reward-related processes such as anticipation, motivation, decision-making, and reward learning^1,2^. In particular, anhedonia may manifest differently across its anticipatory and consummatory dimensions both in anticipation of future events and during present moments. As a transdiagnostic symptom, anhedonia represents a common thread across psychiatric disorders including schizophrenia (SCZ) and major depressive disorder (MDD)^2,3^. Symptoms expressed along a continuous spectrum rather than fitting into discrete diagnostic categories pose challenges for current classification systems^4^. Understanding the commonalities and differences underlying the multidimensional components of anhedonia can significantly enhance our comprehension of transdiagnostic psychopathology in the development of psychiatric disorders.

Meta-analytic studies showed that patients with SCZ and MDD had reduced positive affect in self-report and clinical interview formats^5,6^. Recent lab-based behavioral paradigms (i.e., Monetary Incentive Delay, MID) have objectively measured and disentangled the two components of anhedonia by evaluating affective experience during reward anticipation and consumption^7,8^. Accumulating evidence has demonstrated a phenomenon termed the “liking-wanting anhedonia paradox” in patients with SCZ, characterized by a dissociation between anticipatory anhedonia (reduced “wanting”) and consummatory anhedonia (preserved “liking”)^9^. This paradox refers to the finding that exhibit attenuated pleasure in anticipation to future reward that may lead to decreased motivation for pleasure but intact hedonic capacity as comparable consummatory pleasurable experiences to healthy control (HC) when receiving positive outcomes^10–12^. This dissociation suggests that motivational deficits in SCZ may stem primarily from impairments in anticipatory rather than consummatory pleasure processes. In contrast, MDD patients showed more generalized deficits affecting both anticipatory and consummatory pleasure^13,14^. People with high schizotypal traits (ST) and clinical high-risk populations for psychosis showed another paradox, known as the ‘schizophrenia spectrum anhedonia paradox’, i.e., they exhibited deficits in both anticipatory and consummatory pleasure^15^. Furthermore, research found that the severity of anhedonia could predict the severity of depressive symptoms^16^. Individuals with subthreshold depression (SD) also displayed a significant reduction in affective responses to both positive and negative stimuli^17,18^, particularly evident in anticipatory pleasure^19,20^. Notably, despite existing evidence indicating impairments in two dimensions of anhedonia to varying degrees in SCZ and MDD, the current research had largely focused on a single psychiatric condition, but lacked transdiagnostic samples to clarify whether the manifestations of multidimensional anhedonia would be common or different between the SCZ and the MDD spectrum.

Whilst the traditional models viewed symptoms as manifestations of the underlying disorders, the network approach shifted the focus from disorders to symptoms, and emphasized the intricate relationships among symptoms and how these connections would contribute to psychopathology across various disorders^21–23^. This approach is advantageous for understanding comorbidity and exploring the relationships between symptoms across diagnostic boundaries. Within such framework, symptoms are represented as nodes, while the connections or associations between them are depicted as edges^24^. A notable aspect of symptom networks lies in assessing the significance of nodes within the network structure, often employing centrality metrics to identify key symptoms^25^. Specifically, strength represents the sum of absolute weighted connections, with higher values indicating stronger overall connectivity with other symptoms. Expected influence (EI) accounts for both positive and negative edges by summing signed connection weights, where higher values suggest greater potential to activate other symptoms in the network. Betweenness quantifies how often a node lies on shortest paths between other nodes, with higher values reflecting greater importance in bridging different symptom clusters. Closeness measures the inverse of average distance to other nodes, where higher values indicate a symptom can more rapidly influence or be influenced by others in the network. These metrics can help identify which symptoms are most central to the network and a symptom with high centrality is strongly linked to other symptoms^26,27^. Recent research has underscored the prominent role of anhedonia within symptom networks, with a growing focus on different aspects of anhedonia relate to various psychiatric conditions. For instance, several network analyses have identified pleasure deficits as the central node in the symptom networks of both SCZ and MDD^16,28,29^. Additionally, recent network analysis studies examined anticipatory and consummatory anhedonia in psychiatric disorders, and reported a stronger association between anticipatory pleasure and schizotypal traits compared to consummatory pleasure^30^. However, the interplay between anticipatory and consummatory anhedonia in a transdiagnostic sample with SCZ and MDD patients as well as their respectively subclinical populations remained largely unknown.

This study aimed to investigate the anticipatory and consummatory aspects of anhedonia in a combined sample of SCZ patients, MDD patients, individuals with high ST and SD. We conducted a network analysis to identify the central role of the symptom network and compared the network structures to examine whether the interconnections between various aspects of anhedonia differ between SCZ and MDD. Based on the prior research demonstrating that anticipatory pleasure deficits are more consistently observed across SCZ and MDD than consummatory pleasure deficits^5,31^ and the hypothesis of the network theory, which posits that mental disorders represent a stable state within a strongly connected network^21^, we expected that anticipatory pleasure would be central to the network. Furthermore, we hypothesized that the networks in patients with SCZ and MDD would show stronger connections involving both anticipatory and consummatory anhedonia compared to individuals with high ST and SD, reflecting more pronounced blunted affective responses during both reward anticipation and consumption in clinical populations.

## Methods

### Participants

#### Overview and recruitment

A total of 821 participants were recruited from Shanghai (*n* = 504), Beijing (*n* = 94), from Weifang (*n* = 205), and Hong Kong (*n* = 18). Among the entire sample, 379 participants were derived from previously published samples, including 44 patients with SCZ and 28 patients with MDD as well as 57 individuals with ST, 55 participants with SD, and 195 HC that were derived from previously published samples^32–34^.

#### Clinical sample

The clinical sample included 175 patients (92 females) diagnosed with SCZ (*n* = 115) or MDD (*n* = 60) from the aforementioned mental health centers. All patients with SCZ and MDD who met the diagnostic criteria outlined in the Diagnostic and Statistical Manual of the American Psychiatric Association, Fifth Edition (DSM-5)^35^. Participants were excluded from the study if they met any of the following criteria: (a) having other psychiatric disorders diagnosed using the DSM-5; (b) having a history of neurological illness; (c) low intelligence (IQ < 70); (d) current or a history of drug/substance abuse; or (e) receiving modified electroconvulsive therapy in the previous 6 weeks.

#### Subclinical sample

The subclinical sample consisted of 212 participants (139 females), including 83 with ST, 110 with SD, 19 with both ST and SD, all recruited via open advertisements on social media platforms.

For the ST subgroup, individuals were enrolled if they met the following criteria: (a) scoring 36 or higher on the Schizotypal Personality Questionnaire (SPQ)^36,37^ and (b) having an IQ score of 70 or higher. For the SD subgroup, individuals were included if they met the following criteria: (a) scoring 16 or higher on the Beck Depression Inventory-I (BDI-I)^38^ and (b) having an IQ score of 70 or higher. Additional exclusion criteria included: (a) no current or past history of any DSM-5 disorder; (b) no history of neurological disorder or brain injury; and (c) no current or past history of drug or substance dependence.

#### Healthy controls

The remaining 394 participants served as HC and were recruited via open advertisements on social media platforms. HC were enrolled if they fulfilled the following criteria: (a) a BDI-I score below 16; (b) an SPQ score below 36; (c) no current or past history of any DSM-5 disorder; (d) no history of neurological disorder or brain injury; and (e) no current or past history of drug or substance dependence.

**Fig. 1:**
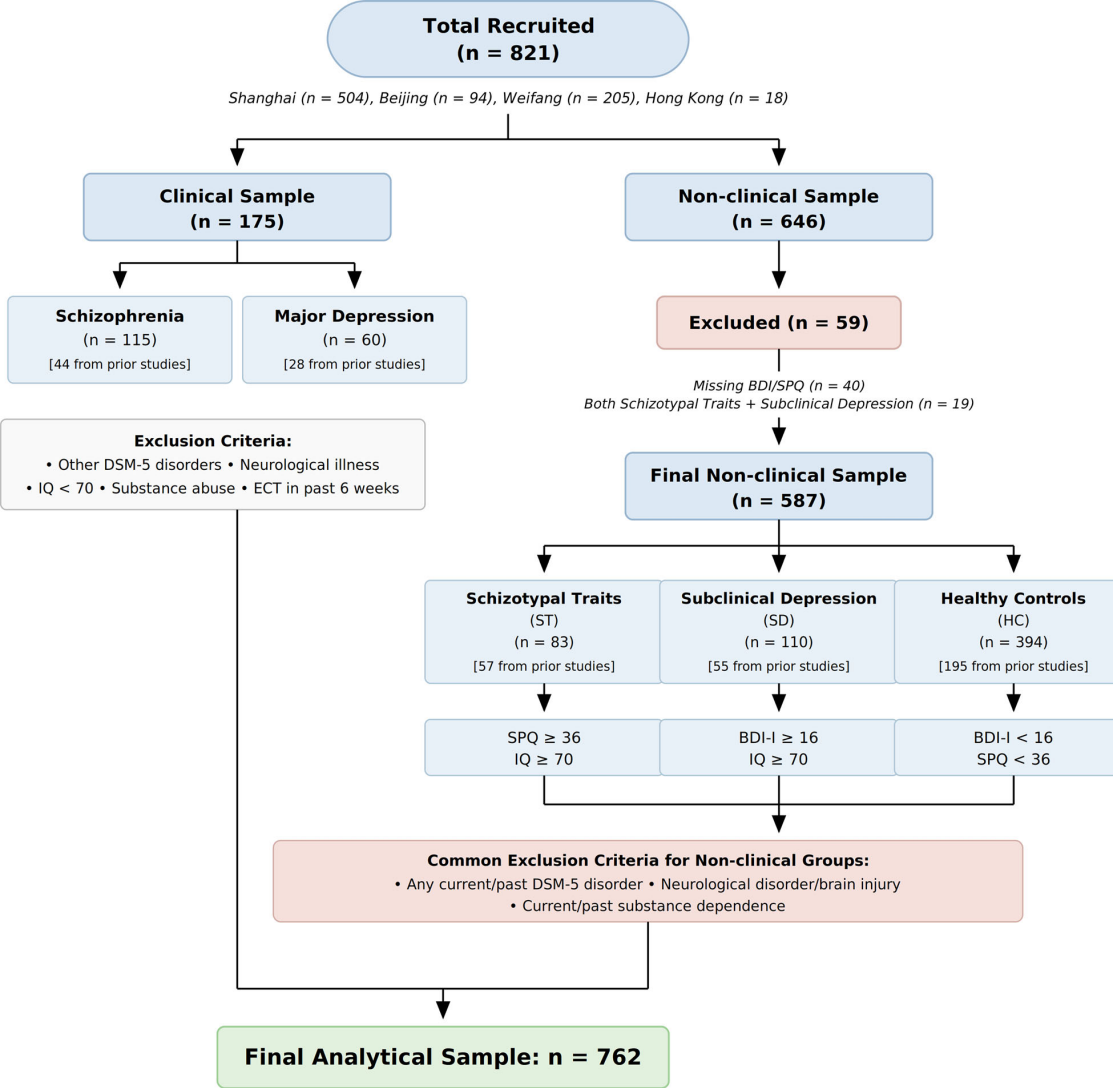
Participant flowchart. Flowchart illustrating the recruitment, exclusion criteria, and derivation of analytical samples. A total of 821 participants were recruited, comprising a clinical sample (*n* = 175; SCZ, *n* = 115; MDD, *n* = 60) and a non-clinical sample (*n* = 646). From the non-clinical sample, 59 participants were excluded due to missing classification data or overlapping subclinical criteria, yielding a final non-clinical sample of 587 participants classified into schizotypal traits (ST, *n* = 83), subclinical depression (SD, *n* = 110), and healthy controls (HC, *n* = 394). The whole-sample network estimation used the full recruited sample (*n* = 821), whereas group-level network comparisons were conducted on the final analytical sample (*n* = 762). Numbers in brackets indicate participants drawn from prior studies. SPQ Schizotypal Personality Questionnaire, BDI-I Beck Depression Inventory-I, IQ intelligence quotient, DSM-5 Diagnostic and Statistical Manual of Mental Disorders, Fifth Edition, ECT electroconvulsive therapy.

The whole-sample network structure estimation was conducted using this full sample (*n* = 821). For the group-level network comparison analyses, participants were classified into clinical and non-clinical subgroups. Among the 646 non-clinical participants, 59 were excluded: 40 due to missing BDI or SPQ data required for subgroup classification, and 19 because they met criteria for both high schizotypal traits and subthreshold depression, precluding unambiguous group assignment. This yielded a final classified sample of 762 participants for the group-level comparisons, comprising 175 clinical patients (SCZ, *n* = 115; MDD, *n* = 60), 193 subclinical individuals (ST, *n* = 83; SD, *n* = 110), and 394 HC. For the transdiagnostic network comparisons, the clinical group (*n* = 175), subclinical group (*n* = 212, comprising ST and SD), and HC group (*n* = 394) were compared. For the spectrum-specific comparisons, analyses were conducted within the schizophrenia spectrum (SCZ, *n* = 115; ST, *n* = 83; HC, *n* = 394) and the depressive spectrum (MDD, *n* = 60; SD, *n* = 110; HC, *n* = 394), respectively. A participant flowchart documenting all exclusions and the derivation of each subsample is presented in Fig. 1 (also see Supplementary Tables S2, S3).

The study was approved by Human Research Protection of Shanghai Mental Health Center (2017-19R), Institute of Psychology, Chinese Academy of Sciences (H18030) and East China Normal University (HR 472-2019). Prior to the commencement of assessments, all participants were required to provide written informed consent.

### Assessments

#### Monetary incentive delay (MID) task

The modified behavioral MID task was utilized to explore anticipatory and consummatory components of anhedonia by evaluating the affective responses in reward processing^39^. During each trial, participants first viewed a cue stimulus (2000 ms) indicating the upcoming condition: a circle signaled potential reward gain (¥0 or ¥5), a square signaled potential reward loss (¥0 or ¥5), and a triangle signaled the neutral control condition (¥0). After the cue, participants rated their subjective valence of anticipatory affect using a 9-point Likert scale. Following the rating, a fixation cross was presented (2000–3000 ms) during the anticipation phase, after which a white star target appeared on the screen. Participants were instructed to press a button as quickly as possible while the target was displayed. The target presentation time was individually adjusted using an adaptive algorithm to maintain an approximate hit rate of 66% across participants, thereby calibrating task difficulty to each individual’s response speed and controlling for individual differences in psychomotor functioning. If the target was successfully hit within the response time interval, participants received monetary rewards or avoided losses; failure to respond in a timely manner resulted in reward omission or monetary loss. Feedback indicating the outcome was then displayed (2000 ms) during the consummatory phase, followed by a subjective valence rating of consummatory affect on a 9-point Likert scale (see Yan et al.^33^ for more described details).

In our study, participants completed either the original or brief (SCZ, *n* = 58; HC, *n* = 109) versions of the MID task. The two versions varied in the magnitude of monetary rewards. In the original version, monetary amounts included ¥0, ¥0.5, and ¥5, whereas in the brief version, participants encountered cues with monetary values of either ¥0 or ¥5. The brief MID task included 60 trials, and the original version included 72 trials. For the current network analyses, we focused on the control condition (¥0) and high-reward condition (¥5), which were common to both task versions. These ratings were averaged for the anticipatory phase, including the control (Anti_C), reward gain (Anti_G), and loss (Anti_L) conditions. Additionally, we assessed consummatory affective responses for no monetary win or loss (Cons_C/Cons_NC), reward gain (Cons_G)/omission (Cons_NG), and reward loss (Cons_L)/loss avoidance (Cons_NL). For inpatients who completed the MID task, tokens were used as monetary incentives, while all other participants received actual monetary compensation.

#### Clinical symptoms assessment and self-reported scales

The Positive and Negative Syndrome Scale (PANSS) was utilized to evaluate the severity of schizophrenia symptoms, as it included three sub-scales covering general psychopathology, positive, and negative symptoms^40^. To assess the severity of depressive symptoms in MDD patients, the Chinese version of the Hamilton Depression Scale (HAMD) was employed, which has been shown to be a reliable and valid measure for detecting depression^41,42^.

Additionally, we adopted the modified Chinese version of the BDI, which was a widely used self-rating scale with 21 items on a 4-point scale from 0 to 3 for assessing the severity of depression^38^. To evaluate schizotypal traits, we utilized the Chinese version of the SPQ, which encompasses three factors: cognitive-perceptual, interpersonal, and disorganization^36,37^.

### Data preparation and analyses

#### Group differences in demographic characteristics and clinical symptoms

We compared demographic characteristics and clinical variables among groups using univariate analysis of variance (ANOVA). The distribution of sex was analyzed using a Chi-square test, with a significance level set at *p* < .05.

#### Group differences in reaction times

To examine whether group differences in consummatory affective ratings could be confounded by psychomotor slowing or inattention, we analyzed reaction times (RTs) during the consummatory phase of the MID task. Mixed-design ANOVAs with Group as a between-subjects factor and MID Condition (gain, loss, neutral) as a within-subjects factor were performed on RTs for the overall sample (clinical, subclinical, and HC), the schizophrenia spectrum (SCZ, ST, and HC), and the depressive spectrum (MDD, SD, and HC), respectively (see Supplementary Materials for full results).

#### Network structure estimation and comparison

The network structure for the whole sample (*n* = 821) was estimated with anticipatory and consummatory affective ratings as nodes. Partial Spearman correlation coefficient for each pair of nodes was calculated while adjusting for all the other variables as the strength of the edge between two nodes (i.e., edge weight). A Gaussian graphical model (GGM) was fitted using graphical Least Absolute Shrinkage and Selection Operator (LASSO) with the extended Bayesian information criterion (EBICglasso algorithm) and tuning hyperparameter was set at 0.5 to obtain a network with high specificity and stability^43^. The network visualization was presented by the Fruchterman-Reingold algorithm to identify and place central measures (i.e., highly correlated nodes) in the network^44^. To assess the predictability of a node, we calculated the proportion of variance within each node that is explained by its connections to other nodes in the network. Additionally, we estimated the network centrality (i.e., strength, closeness, betweenness and EI) to evaluate the importance of nodes in the network. We also conducted the network accuracy and stability analysis (Supplementary Figs. S5, S6). The construction of networks, calculation of centrality indices, and visualization were carried out using the R packages ‘bootnet’ (https://cran.r-project.org/package=bootnet; v.1.5.0) and ‘mgm’ (https://cran.r-project.org/package=mgm; v.1.2.13).

We compared the edge weights and centrality indices across the clinical patients with SCZ or MDD (*n* = 175), subclinical individuals with ST or SD (*n* = 212), and HC (*n* = 394) samples using the ‘NetworkComparisonTest’ package in R (https://cran.r-project.org/web/packages/NetworkComparison Test/index.html; v.2.2.1)^45^. Two-tailed permutation tests with 10,000 times were performed to estimate the invariance of network structures (the element-wise difference in all the edges), global strengths (an overall network connectivity) and edge weights^46^ among the clinical, subclinical, and HC groups. We focused on the edges between anticipatory affect and consummatory affect metrics and Benjamini–Hochberg (BH) false discovery rate (FDR) correction was used for multiple comparisons^47^.

Given that the RT analyses revealed significant group differences in consummatory-phase RTs (see Supplementary Results and Supplementary Table S1), we performed additional network analyses controlling for potential confounds. Specifically, for each group, we regressed sex, age, and RTs from the three MID conditions (neutral, gain, and loss) from each node variable using linear regression, and then re-estimated the networks using the resulting residuals. Network Comparison Tests were subsequently repeated on these covariate-adjusted networks using identical parameters. To test the robustness of partial correlation findings, we also re-estimated all networks using zero-order correlations^48^ (Supplementary Figs. S2–S4).

Furthermore, to identify the commonalities and differences underlying anhedonia between disorders, we performed separate network comparisons within the schizophrenia spectrum (*n* = 198), consisting of the SCZ (*n* = 115), ST (*n* = 83), and HC (*n* = 394) groups, as well as within the depressive spectrum (*n* = 170), comprising the MDD (*n* = 60), SD (*n* = 110), and HC (*n* = 394) groups. The same covariate-controlled analyses were also conducted for these spectrum-specific comparisons. All analyses were conducted using R version 4.2.0, and statistical significance was defined as *p* < 0.05.

## Results

### Demographic characteristics and clinical symptoms

Table 1 presents the demographic and clinical characteristics of the three groups. The groups differed significantly in sex distribution (χ² (2) = 7.98, *p* = 0.020, φ = 0.15), with a more balanced male-to-female ratio in the clinical group (83/92) compared to the subclinical (73/139) and HC (175/219) groups. The clinical group was significantly older (*M* = 30.49, *SD* = 8.67) than the subclinical (*M* = 19.72, *SD* = 5.25) and HC (*M* = 20.08, *SD* = 5.59) groups (*F* (2, 778) = 188.36, *p* < 0.001, η² = 0.33), whereas years of education did not differ significantly across groups (*F* (2, 778) = 1.10, *p* = 0.332, η² = 0.00).

**Table 1:** Descriptive statistic of demographic and clinical symptoms data among the participants.

	Clinical (*n* = 175)	Subclinical (*n* = 212)	HC (*n* = 394)	*χ²/F*	*p*	*φ/η2*
Sex (male/female)	83/92	73/139	175/219	7.98	0.020	0.15
Age	30.49 (8.67)	19.72 (5.25)	20.08 (5.59)	188.36	<0.001	0.33
Education (years)	13.21 (3.01)	12.88 (2.53)	12.84 (2.99)	1.10	0.332	0.00
BDI	18.15 (12.91)	18.26 (9.28)	5.99 (4.58)	168.83	<0.001	0.36
SPQ	32.20 (29.09)	41.69 (9.32)	19.63 (9.36)	186.9	<0.001	0.52
DOI (month)	35.37 (59.09)					
Dosage of drug for SCZ (Chlorpromazine, mg/d)	289.42 (206.75)					
Dosage of drug for MDD (Fluoxetine equivalence, mg/d)	10.10 (16.25)					

The clinical group was included schizophrenia (SCZ) and major depressive disorder (MDD). The subclinical group was comprised individuals with schizotypal traits (ST) and subclinical depression (SD).

*DOI* Duration of Illness, *BDI* Beck Depression Inventory, *SPQ* Schizotypal Personality Questionnaire, *SCZ* schizophrenia, *MDD* major depressive disorder.

Regarding clinical symptom measures, both the clinical (*M* = 18.15, *SD* = 12.91) and subclinical (*M* = 18.26, *SD* = 9.28) groups reported significantly higher BDI scores than the HC group (*M* = 5.99, *SD* = 4.58; *F* (2, 778) = 168.83, *p* < 0.001, η² = 0.36). SPQ scores also differed significantly across groups (F (2, 778) = 186.90, *p* < 0.001, η² = 0.52), with the subclinical group reporting the highest scores (*M* = 41.69, *SD* = 9.32), followed by the clinical group (*M* = 32.20, *SD* = 29.09) and the HC group (*M* = 19.63, *SD* = 9.36).

### Network structures for the whole sample

**Fig. 2:**
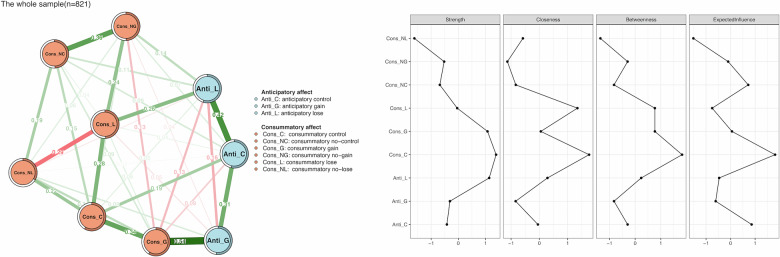
A network structure containing the anticipatory and consummatory affect for the entire sample. Nodes in blue signify anticipatory affect, while nodes in orange signify consummatory affect. The lines (edge weights) depict the strength of association between nodes, with green edges indicating positive associations and red edges representing negative associations. Thicker lines indicate stronger connections between nodes. Additionally, the centrality plots provide insight into standardized node strength, closeness, betweenness, and expected influence within the network. Anti_C anticipatory affect under the control condition, Anti_G anticipatory affect under the gain condition, Anti_L anticipatory affect under the lose condition, Cons_C consummatory affect when hitting the target under the control condition, Cons_NC consummatory affect when missing the target under the control condition, Cons_G consummatory affect under the gain condition, Cons_NG consummatory affect towards reward omission, Cons_L consummatory affect under the lose condition, Cons_NL consummatory affect towards loss avoidance.

We visualized the overall network structure between anticipatory and consummatory affects, along with the centrality indexes of the network, for the entire sample of 821 participants (See Fig. 2). Regarding anticipatory affect, a negative correlation emerged between the subjective ratings while anticipating reward gain and those while anticipating reward loss (Anti_G--Anti_L). In terms of consummatory affect, similar negative associations were noted between responses during reward gain and reward omission (Cons_G--Cons_NG), and between responses during loss and loss avoidance (Cons_L--Cons_NL). Additionally, we found strong connections between anticipatory and consummatory affects. Specifically, there was a positive correlation between anticipatory and consummatory responses in the context of reward gain (Anti_G--Cons_G), whereas negative correlations emerged between consummatory responses during reward gain and anticipatory responses for reward loss (Cons_G--Anti_L). As shown in Fig. 2, anticipatory affect (Anti_C, EI = 1.018) and consummatory affect (Cons_C, EI = 1.262) exhibited the highest centrality indexes under the control condition (see also Supplementary Figs. S1, S2). Node predictability ranged from 33.4% (Cons_NL) to 53.4% (Anti_C), with an average predictability of 51.0%. After controlling for sex, age, and RTs from the three MID conditions, the overall network structure and centrality pattern remained largely unchanged (Supplementary Fig. S2), with node predictability ranging from 33.6% (Cons_NL) to 52.8% (Anti_C).

### Network comparisons in the clinical, subclinical and HC group

**Fig. 3:**
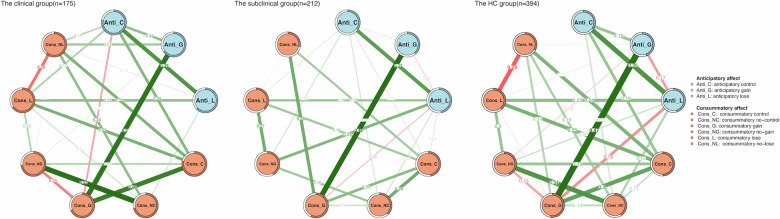
The network structures depicting anticipatory and consummatory affect in the clinical, subclinical, and HC samples. Nodes in blue symbolize anticipatory affect, while nodes in orange represent consummatory affect. The lines (edge weights) illustrate the strength of association between nodes, with green edges denoting positive associations and red edges indicating negative associations. Thicker lines indicate stronger connections between nodes.

We constructed separate networks for anticipatory and consummatory affect in the clinical, subclinical, and HC groups, as depicted in Fig. 3. The network invariance test comparing the structural differences between the clinical patients and subclinical individuals did not yield statistical significance (M = 0.321, *p* = 0.167). However, the invariance test for global strength (GS) was weaker for the network of subclinical group compared to clinical group (S = 1.509, *p* = 0.049; GS clinical = 4.838, GS subclinical = 3.329). Interestingly, we found a marginally significant difference between the subclinical and HC groups in the invariance test of network structure (M = 0.304, *p* = 0.076). Furthermore, the global strength invariance test revealed a significant difference between the subclinical and HC group, indicating a weaker network connection within the subclinical group compared to the HC group (S = 1.561, *p* = 0.013; GS _HC_ = 4.890, GS subclinical = 3.329). No significant difference was observed in the invariance tests for edge weights after the FDR correction. No significant differences were found between the clinical and HC group regarding network structure invariance (M = 0.191, *p* = 0.792), global strength (S = 0.052, *p* = 0.935), or edge weights (FDR correction, *p*s > 0.3). Concerning centrality indexes, consummatory affect under the control condition (i.e., Cons_C) exhibited the highest node strength and EI centrality in both the clinical and HC groups, as illustrated in Supplementary Fig. S3.

After controlling for sex, age, and reaction times, the network comparison results were partially attenuated. The network structure invariance tests remained non-significant for all three pairwise comparisons (clinical vs. subclinical: M = 0.308, *p* = 0.172; clinical vs. HC: M = 0.189, *p* = 0.792; subclinical vs. HC: M = 0.268, *p* = 0.186). Notably, the previously significant global strength difference between the clinical and subclinical groups became non-significant after covariate adjustment (S = 0.740, *p* = 0.386; GS _clinical_ = 4.563, GS _subclinical_ = 3.823), as did the difference between the subclinical and HC groups, which showed a marginal trend after adjustment (S = 1.099, *p* = 0.072; GS _subclinical_ = 3.823, GS _HC_ = 4.922). The comparison between the clinical and HC groups remained non-significant (S = 0.359, *p* = 0.592). No significant differences were observed for edge weights in any pairwise comparison after FDR correction.

### Network comparisons in the schizophrenia and depression spectrum

Interestingly, we observed significant difference for the network structure between the group with patients with MDD and SD subgroups (*n* = 170) and the HC group (*n* = 394) (M = 0.438, *p* = 0.002). However, invariance tests for global strength (S = 0.314, *p* = 0.704; GS _depressive spectrum_ = 4.576, GS _HC_ = 4.890), and edge weights (FDR correction, *ps* > 0.9) were non-significant.

In the comparison within the schizophrenia spectrum, no significant differences were observed in network structure between patients with SCZ and ST subgroups (*n* = 198) and HC (*n* = 394) groups (M = 0.203, *p* = 0.632). here were no significant differences in global strength (S = 0.868, *p* = 0.190; GS _schizophrenia spectrum_ = 5.758, GS _HC_ = 4.890) or edge weights (FDR correction, *p*s > 0.7).

**Fig. 4:**
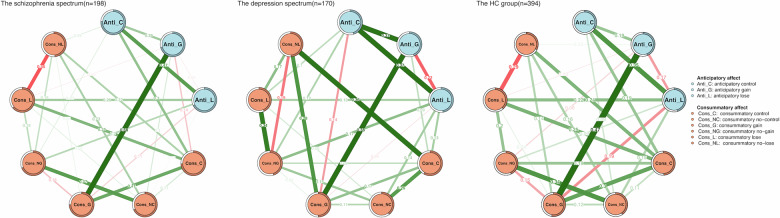
The network structures for the anticipatory and consummatory affect in the schizophrenia and depression spectrum and HC group. Blue nodes represent anticipatory affect. Orange nodes represent consummatory affect. The lines (edge weights) represent the strength of association between the nodes. Green edges indicate positive associations and red edges represent negative associations. Thicker lines indicate stronger connections between the nodes.

Finally, we compared network structures between patients in the schizophrenia spectrum and those in the depression spectrum. The invariance test for network structure showed a significant difference (M = 0.551, *p* < 0.001). However, no significant difference was found in global strength between the two networks (S = 1.182, *p* = 0.135). We observed notably stronger edge weights for subjective ratings to reward gain during both anticipatory and consummatory phases (i.e., Anti_G-Cons_G) in SCZ patients and ST individuals compared to MDD patients and SD individuals (*E* = 0.304, *p* = 0.032; edge weights: the schizophrenia spectrum = 0.658, the depression spectrum = 0.354). However, no other significant difference was found for edge weights (FDR correction, *p*s > 0.2). (Please see Fig. 4).

After controlling for covariates, the spectrum-specific comparisons yielded broadly consistent results. The significant difference in network structure between the schizophrenia and depression spectra was preserved (M = 0.500, *p* < .001). However, the previously significant edge weight difference for the Anti_G-Cons_G edge was attenuated to marginal significance after covariate adjustment (*p* = 0.073). The network structure difference between the depressive spectrum and the HC group, as well as the non-significant comparisons involving the schizophrenia spectrum and HC group, remained unchanged after controlling for covariates. Detailed results of the covariate-controlled analyses are presented in Supplementary Results (also see Supplementary Figs. S2–S4).

## Discussion

This study used transdiagnostic network analysis to investigate the interactions of anhedonia in multifaceted dimensions based on a sample of SCZ, MDD and their subclinical counterparts. The findings showed the affective response under the neutral outcome played the most central role in the network. Further network comparisons revealed that individuals with ST or SD had lower global strength in anticipatory and consummatory affective responses compared to patients with SCZ or MDD and HC. Additionally, SCZ patients and individuals with ST exhibited stronger connections for pleasurable affective responses during both reward anticipation and consumption compared to those with MDD or SD.

Our centrality analysis of different anhedonia components revealed that the consummatory affective response toward the neutral condition was central node across the transdiagnostic network, as well as in both clinical and subclinical network analyses. This consummatory affective pattern plays an important role on affective responses to both reward gains and losses, as evidenced by stronger direct interconnections to anticipatory and consummatory components of anhedonia. Given its central role in the network, the affective response to neutral cues may serve as a baseline for calibrating affective responses to other stimuli, thereby influencing both reward anticipation and goal-directed behavior. This finding may reflect the interaction of multiple underlying mechanisms across different diagnostic spectra. From one perspective, the aberrant salience hypothesis offers an important explanatory framework, particularly within the SCZ spectrum^49^. Due to dysfunction in the dopamine system, patients with SCZ may attribute excessive significance to neutral or irrelevant stimuli, which in turn enhances their affective salience to neutral cues, potentially increasing their centrality within the symptom network. For instance, research found that both patients with SCZ and individuals with psychotic-like experiences have shown heightened responses to neutral stimuli^50^. On the other hand, the Evaluative Space Model (ESM) provides a complementary explanation that may apply to both SCZ^51,52^ and MDD^53^. This model posits two distinct emotional systems: a positive system that generates a positivity offset—that is, greater positive than negative affect in response to low-arousal or neutral stimuli—and a negative system that produces a negativity bias under high-arousal conditions^54^. In healthy individuals, these systems are typically balanced; however, in psychiatric populations, this balance may be disrupted. Specifically, individuals with SCZ or MDD may exhibit a reduced positivity offset or enhanced negativity bias, leading to blunted or negative affective responses to neutral cues. Such affective dysregulation may increase the functional importance of responses to neutral stimuli in the emotional system and explain their central role in anhedonia-related symptom networks. Taken together, the centrality of consummatory responses to neutral stimuli may reflect both aberrant salience attribution (i.e., increased responsiveness to irrelevant cues) and a reduction in positivity offset (i.e., diminished positive affect under neutral conditions), thus offering a transdiagnostic mechanism underlying dysfunctional emotional processing.

Our transdiagnostic network findings revealed that individuals with ST and SD exhibited significantly lower global network strength compared to those with SCZ and MDD, indicating weaker connections between hedonic experiences and motivation for pleasure. The finding supported the clinical staging model and the vulnerability hypothesis of network theory, which propose that as severity increases, the connectivity between symptoms becomes more specific and stable, leading to stronger interrelationships among symptoms and the activation of other symptoms^21,55^. This indicated a continuum in affective response patterns from subclinical to clinical levels, with heightened symptom severity potentially linked to impaired affective processing and closer interconnections between different hedonic components. Notably, after controlling for sex, age, and consummatory-phase RTs, the previously observed difference in global network strength between the clinical and subclinical groups no longer statistically significant. This suggests that the weaker network connectivity observed in subclinical groups may be partially attributable to demographic and psychomotor factors. Nevertheless, the direction and pattern of the original findings were preserved after covariate adjustment, and the subclinical group consistently showed the lowest global strength across all analyses, lending support to the broader interpretation that hedonic components at the subclinical stage are less tightly coupled. Our network approach aligns with the RDoC positive valence systems framework by quantifying the overall strength of associations among anticipatory and consummatory pleasure components across diagnostic categories. The relatively weaker integration observed in individuals with ST and SD, even after accounting for potential confounds, suggests that the functional coupling between reward anticipation and outcome evaluation may already be compromised prior to the onset of full-threshold psychopathology. Importantly, unlike categorical diagnostic classifications, global strength varies continuously across individuals, making it a potential indicator of subtle alterations in hedonic network integrity that may precede clinically recognized impairment, thereby facilitating earlier identification of individuals at elevated risk for progression to clinical anhedonia. Longitudinal research is needed to determine whether reduced global strength prospectively predicts the emergence of clinical anhedonia over time. Additionally, similarities observed between clinical and subclinical network structures aligned with previous research^56,57^, and suggested that connectivity patterns in affective responses remain consistent across different levels of severity. This may imply that these disorders share common mechanisms in symptom expression or interactions. Given the heterogeneity of research outcomes, it is important to note that previous studies have included diverse psychiatric disorders with varying severities^56^. Future research should consider longitudinal data to construct contemporaneous and temporal symptom networks^58^, which could provide deeper insights into the developmental psychopathology of anhedonia.

The disparities in network structure between the spectra substantiated our hypothesis, revealing a stronger connection specifically between anticipatory and consummatory pleasure in SCZ patients and ST individuals compared to MDD patients and SD individuals. The heightened connectivity implies that the emergence of one symptom may lead to changes in others, contributing to the formation of a more robust network of associations^21^. This finding indicated a more complex interaction among dimensional anhedonia components, potentially facilitating a broader spread of affective responses within the schizophrenia spectrum. Importantly, the significant difference in network structure between the schizophrenia and depression spectra was preserved after controlling for covariates, confirming that these structural disparities are not driven by group differences in psychomotor speed or demographic characteristics. This phenomenon aligned with the second paradox known as the ‘schizophrenia spectrum anhedonia paradox’^15^, concerning those with ST and clinical high-risk populations for psychosis characterized by experiences resembling symptoms of SCZ^59^ and exhibited deficits in both anticipatory and consummatory pleasure^5,60^. Consequently, anhedonia within the schizophrenia spectrum may manifest as more pervasive abnormalities in affective processing during reward anticipation and consumption compared to the depression spectrum. The interaction between anticipatory and consummatory pleasure may thus represent a core feature of anhedonia across psychiatric disorders, potentially elucidating the differences in psychopathological mechanisms between these disorders.

### Limitation

The current study has several limitations that need to be noted. First, given the limited sample size of individuals with MDD and SCZ available for constructing a relatively stable separate networks related to anticipatory and consummatory affect, there was a high likelihood of overlooking unique and shared patterns of affective responses during reward processing for those patients with different clinical conditions. Second, although participants were recruited from multiple sites (Shanghai, Beijing, Weifang, and Hong Kong) with identical task procedures and diagnostic protocols, we were unable to conduct formal site-level network comparisons. When stratified by both site and diagnostic group, the resulting cell sizes were too small (e.g., Hong Kong, *n* = 18) to support stable network estimation, precluding meaningful cross-site network comparison tests. While the standardized methodology across sites mitigates concerns about procedural variability, we cannot entirely rule out the influence of site-related factors (e.g., regional or clinical setting differences) on the observed network structures. Future studies with larger and more balanced site-specific samples should formally examine the cross-site invariance of anhedonia-related networks. Third, while our MID task primarily utilized monetary rewards, research suggested that social rewards might offer a more precise understanding psychopathological mechanisms of anhedonia across different psychiatric disorders^61^. Therefore, future studies incorporating social rewards can offer additional insights into the transdiagnostic features of anhedonia. Finally, a recent focus on micro-level momentary affective states to understand the nature of psychiatric disorders was proposed by the Momentary Affect Dynamics (MAD) network theory^62^. It linked multiple affects and patterns of behavior towards various contexts and assumed that negative circle of affective states interactions underlie the development of psychiatric disorders, as evidenced by time-series study on affective response in daily life situations with the experience sampling methodology^63,64^. Therefore, future research can explore patterns of change in affect over time by constructing dynamic network models based on individuals’ daily affective experiences.

## Conclusion

Our findings reveal that consummatory affective responses to neutral stimuli in reward processing play a crucial role in the transdiagnostic network. The reduced interaction among affective responses in subclinical individuals, such as those with ST or SD, may predispose them to developing clinical levels of anhedonia observed in patients with SCZ or MDD. Moreover, the intricate relationship between anticipatory and consummatory pleasure has the potential to illuminate the distinct mechanisms underlying anhedonia in SCZ and MDD. These findings emphasize the critical role of multidimensional anhedonia in comprehending the continuum from subclinical to clinical levels of anhedonia across diverse psychiatric disorders, providing valuable insights for future transdiagnostic diagnostics and therapeutic interventions.

## Supplementary information

Supplementary Information

## Data Availability

The anonymized data and analysis code that support the findings of this study are available from the corresponding author upon reasonable request.

## References

[1] Strauss, G. P. & Gold, J. M. A new perspective on anhedonia in schizophrenia. *Am. J. Psychiatry* **169**, 364–373 (2012). 22407079 10.1176/appi.ajp.2011.11030447 PMC3732829

[2] Shankman, S. A. et al. The different facets of anhedonia and their associations with different psychopathologies. In *Anhedonia: A Comprehensive Handbook Volume I: Conceptual Issues And Neurobiological Advances* (ed. Ritsner, M. S.) 3–22 (Springer Netherlands, Dordrecht, 2014). 10.1007/978-94-017-8591-4_1.

[3] Kieslich, K., Valton, V. & Roiser, J. P. Pleasure, Reward Value, Prediction Error And Anhedonia. In *Anhedonia: Preclinical, Translational, and Clinical Integration* (ed. Pizzagalli, D. A.) 281–304 (Springer International Publishing, Cham, 2022). 10.1007/7854_2021_295.

[4] Krueger, R. F. & Markon, K. E. A dimensional-spectrum model of psychopathology: progress and opportunities. *Arch. Gen. Psychiatry* **68**, 10–11 (2011). 21199961 10.1001/archgenpsychiatry.2010.188

[5] Hallford, D. J. & Sharma, M. K. Anticipatory pleasure for future experiences in schizophrenia spectrum disorders and major depression: a systematic review and meta-analysis. *Br. J. Clin. Psychol.* **58**, 357–383 (2019). 30854671 10.1111/bjc.12218

[6] Visser, K. F., Chapman, H. C., Ruiz, I., Raugh, I. M. & Strauss, G. P. A meta-analysis of self-reported anticipatory and consummatory pleasure in the schizophrenia-spectrum. *J. Psychiatr. Res.* **121**, 68–81 (2020). 31783235 10.1016/j.jpsychires.2019.11.007 PMC6939125

[7] Knutson, B. & Heinz, A. Probing psychiatric symptoms with the monetary incentive delay task. *Biol. Psychiatry* **77**, 418–420 (2015). 25645271 10.1016/j.biopsych.2014.12.022

[8] Lutz, K. & Widmer, M. What can the monetary incentive delay task tell us about the neural processing of reward and punishment? *Neurosci. Neuroecon.* **3**, 33–45 (2014).

[9] Strauss, G. P. The emotion paradox of anhedonia in schizophrenia: or is it? *Schizophr. Bull.* **39**, 247–250 (2013). 23328158 10.1093/schbul/sbs192 PMC3576151

[10] Frost, K. H. & Strauss, G. P. A review of anticipatory pleasure in schizophrenia. *Curr. Behav. Neurosci. Rep.* **3**, 232–247 (2016). 27980891 10.1007/s40473-016-0082-5 PMC5152682

[11] Gold, J. M. et al. Negative symptoms of schizophrenia are associated with abnormal effort-cost computations. *Biol. Psychiatry* **74**, 130–136 (2013). 23394903 10.1016/j.biopsych.2012.12.022 PMC3703817

[12] Saleh, Y., Jarratt-Barnham, I., Fernandez-Egea, E. & Husain, M. Mechanisms underlying motivational dysfunction in schizophrenia. *Front. Behav. Neurosci*. **15**, 709753 (2021). 10.3389/fnbeh.2021.709753 PMC8460905 34566594

[13] Rzepa, E., Fisk, J. & McCabe, C. Blunted neural response to anticipation, effort and consummation of reward and aversion in adolescents with depression symptomatology. *J. Psychopharmacol.* **31**, 303–311 (2017). 28093022 10.1177/0269881116681416

[14] Wu, H. et al. Anticipatory and consummatory pleasure and displeasure in major depressive disorder: an experience sampling study. *J. Abnorm. Psychol.* **126**, 149–159 (2017). 27936838 10.1037/abn0000244 PMC5305427

[15] Strauss, G. & Cohen, A. The schizophrenia spectrum anhedonia paradox. *World Psychiatry* **17**, 221–222 (2018). 29856563 10.1002/wps.20529 PMC5980495

[16] Guineau, M. G. et al. Anhedonia as a transdiagnostic symptom across psychological disorders: a network approach. *Psychol. Med.* **53**, 3908–3919 (2023). 35348051 10.1017/S0033291722000575 PMC10317820

[17] Bylsma, L. M. Emotion context insensitivity in depression: toward an integrated and contextualized approach. *Psychophysiology* **58**, e13715 (2021). 33274773 10.1111/psyp.13715 PMC8097691

[18] von Klipstein, L. et al. Increased affective reactivity among depressed individuals can be explained by floor effects: an experience sampling study. *J. Affect. Disord.* **334**, 370–381 (2023). 37150221 10.1016/j.jad.2023.04.118

[19] Pu, J. et al. Differential manifestations of anhedonia in people with social anhedonia and subsyndromal depression. *Asian J. Psychiatry* **100**, 104188 (2024). 10.1016/j.ajp.2024.104188 39089075

[20] Rizvi, S. J., Pizzagalli, D. A., Sproule, B. A. & Kennedy, S. H. Assessing anhedonia in depression: potentials and pitfalls. *Neurosci. Biobehav. Rev.* **65**, 21–35 (2016). 26959336 10.1016/j.neubiorev.2016.03.004 PMC4856554

[21] Borsboom, D. A network theory of mental disorders. *World Psychiatry* **16**, 5–13 (2017). 28127906 10.1002/wps.20375 PMC5269502

[22] Epskamp, S., Maris, G., Waldorp, L. J. & Borsboom, D. Network psychometrics. In *The Wiley Handbook of Psychometric Testing*, 953–986 (John Wiley & Sons, Ltd, 2018). 10.1002/9781118489772.ch30.

[23] McNally, R. J. Network analysis of psychopathology: controversies and challenges. *Annu. Rev. Clin. Psychol.* **17**, 31–53 (2021). 33228401 10.1146/annurev-clinpsy-081219-092850

[24] Bringmann, L. F. et al. What do centrality measures measure in psychological networks? *J. Abnorm. Psychol.* **128**, 892–903 (2019). 31318245 10.1037/abn0000446

[25] Haslbeck, J. M. B. & Fried, E. I. How predictable are symptoms in psychopathological networks? A reanalysis of 18 published datasets. *Psychol. Med.* **47**, 2767–2776 (2017). 28625186 10.1017/S0033291717001258

[26] Borsboom, D. & Cramer, A. Network analysis: an integrative approach to the structure of psychopathology. *Annu. Rev. Clin. Psychol.* **9**, 91–121 (2013). 23537483 10.1146/annurev-clinpsy-050212-185608

[27] McNally, R. J. Can network analysis transform psychopathology? *Behav. Res. Ther.* **86**, 95–104 (2016). 27424882 10.1016/j.brat.2016.06.006

[28] Abplanalp, S. J. et al. Understanding connections and boundaries between positive symptoms, negative symptoms, and role functioning among individuals with schizophrenia: a network psychometric approach. *JAMA Psychiatry* **79**, 1014–1022 (2022). 35976655 10.1001/jamapsychiatry.2022.2386 PMC9386606

[29] Hu, H. et al. A transdiagnostic network analysis of motivation and pleasure, expressivity and social functioning. *Nat. Ment. Health* **1**, 586–595 (2023).

[30] Alfimova, M. V., Lezheiko, T., Plakunova, V. & Golimbet, V. Relationships between schizotypal features, trait anticipatory and consummatory pleasure, and naturalistic hedonic States. *Motiv. Emot.* **45**, 649–660 (2021).

[31] Lambert, C. et al. Anhedonia in depression and schizophrenia: a transdiagnostic challenge. *CNS Neurosci. Ther.* **24**, 615–623 (2018). 29687627 10.1111/cns.12854 PMC6489811

[32] Sun, C. et al. Emotion Context Insensitivity is generalized in individuals with major depressive disorder but not in those with subclinical depression. *J. Affect. Disord.* **313**, 204–213 (2022). 35777495 10.1016/j.jad.2022.06.069

[33] Yan, C. et al. Anticipatory pleasure for future rewards is attenuated in patients with schizophrenia but not in individuals with schizotypal traits. *Schizophr. Res.* **206**, 118–126 (2019). 30545761 10.1016/j.schres.2018.12.003

[34] Guo, X. et al. Altered empathy correlates with reduced social and non-social reward anticipation in individuals with highÿsocial anhedonia. *PsyCh J.* **12**, 92–99 (2023). 36058882 10.1002/pchj.592

[35] Association, A. Psychiatric. Diagnostic and statistical manual of mental disorders. https://public.ebookcentral.proquest.com/choice/publicfullrecord.aspx?p=1811753 (2013).

[36] Chen, W. J., Hsiao, C. K. & Lin, C. C. H. Schizotypy in community samples: the three-factor structure and correlation with sustained attention. *J. Abnorm. Psychol.* **106**, 649–654 (1997). 9358696 10.1037//0021-843x.106.4.649

[37] Raine, A. The SPQ: a scale for the assessment of schizotypal personality based on DSM-III-R criteria. *Schizophr. Bull.* **17**, 555–564 (1991). 1805349 10.1093/schbul/17.4.555

[38] Beck, A. T. & Beamesderfer, A. Assessment of depression: the depression inventory. In *Psychological measurements in psychopharmacology*, 267–267 (S. Karger, Oxford, England, 1974). 10.1159/000395074. 10.1159/000395074 4412100

[39] Nielsen, L., Knutson, B. & Carstensen, L. L. Affect dynamics, affective forecasting, and aging. *Emotion* **8**, 318–330 (2008). 18540748 10.1037/1528-3542.8.3.318 PMC2652507

[40] Kay, S. R., Fiszbein, A. & Opler, L. A. The positive and negative syndrome scale (PANSS) for schizophrenia. *Schizophr. Bull.* **13**, 261–276 (1987). 3616518 10.1093/schbul/13.2.261

[41] HAMILTON, M. A rating scale for depression. *J. Neurol. Neurosurg. Psychiatry* **23**, 56–62 (1960). 14399272 10.1136/jnnp.23.1.56 PMC495331

[42] Zheng, Y. et al. Validity and reliability of the Chinese Hamilton depression rating scale. *Br. J. Psychiatry* **152**, 660–664 (1988). 3167442 10.1192/bjp.152.5.660

[43] Foygel, R. & Drton, M. Extended Bayesian information criteria for Gaussian graphical models. In *Advances in Neural Information Processing Systems*, 23 (Curran Associates, Inc., 2010).

[44] Epskamp, S., Borsboom, D. & Fried, E. I. Estimating psychological networks and their accuracy: a tutorial paper. *Behav. Res. Methods* **50**, 195–212 (2018). 28342071 10.3758/s13428-017-0862-1 PMC5809547

[45] van Borkulo, C. D. et al. Comparing network structures on three aspects: a permutation test. *Psychol. Methods* **28**, 1273–1285 (2022). 10.1037/met0000476 35404628

[46] van Borkulo, C. D. et al. A new method for constructing networks from binary data. *Sci. Rep.* **4**, 5918 (2014). 25082149 10.1038/srep05918 PMC4118196

[47] Benjamini, Y. & Hochberg, Y. Controlling the false discovery rate: a practical and powerful approach to multiple testing. *J. R. Stat. Soc. Ser. B Methodol.* **57**, 289–300 (1995).

[48] Rose, L., Lynam, D. R. & Miller, J. D. Perils of partialing: can scholars predict residualized variables’ nomological nets? *J. Pers*. **94**, 199–206 (2026). 10.1111/jopy.13024 40195627

[49] Howes, O. D. & Nour, M. M. Dopamine and the aberrant salience hypothesis of schizophrenia. *World Psychiatry* **15**, 3–4 (2016). 26833595 10.1002/wps.20276 PMC4780291

[50] Howes, O. D., Hird, E. J., Adams, R. A., Corlett, P. R. & McGuire, P. Aberrant salience, information processing, and dopaminergic signaling in people at clinical high risk for psychosis. *Biol. Psychiatry* **88**, 304–314 (2020). 32430200 10.1016/j.biopsych.2020.03.012

[51] Bartolomeo, L. & Strauss, G. Unpacking the anhedonia paradox across the psychosis continuum: the role of the positivity offset. *J. Emot. Psychopathol.* **1**, 423–439 (2025).

[52] Strauss, G. P., Visser, K. H., Lee, B. G. & Gold, J. M. The positivity offset theory of anhedonia in schizophrenia. *Clin. Psychol. Sci.* **5**, 226–238 (2017). 28497008 10.1177/2167702616674989 PMC5421554

[53] Gollan, J. K. et al. Twice the negativity bias and half the positivity offset: evaluative responses to emotional information in depression. *J. Behav. Ther. Exp. Psychiatry* **52**, 166–170 (2016). 26434794 10.1016/j.jbtep.2015.09.005 PMC5685183

[54] Bartolomeo, L. A., Raugh, I. M. & Strauss, G. P. The positivity offset theory of anhedonia in schizophrenia: evidence for a deficit in daily life using digital phenotyping. *Psychol. Med.* **53**, 6491–6499 (2023). 36722014 10.1017/S0033291722003774 PMC10600929

[55] McGorry, P. & Os, J. van. Redeeming diagnosis in psychiatry: timing versus specificity. *Lancet* **381**, 343–345 (2013). 23351805 10.1016/S0140-6736(12)61268-9

[56] Groen, R. N., Wichers, M., Wigman, J. T. W. & Hartman, C. A. Specificity of psychopathology across levels of severity: a transdiagnostic network analysis. *Sci. Rep.* **9**, 18298 (2019). 31797974 10.1038/s41598-019-54801-y PMC6892855

[57] Imperiale, M. N., Lieb, R., Calkins, M. E. & Meinlschmidt, G. Multimorbidity networks of mental disorder symptom domains across psychopathology severity levels in community youth. *J. Psychiatr. Res.* **141**, 267–275 (2021). 34265564 10.1016/j.jpsychires.2021.07.010

[58] van der Tuin, S. et al. Dynamic symptom networks across different at-risk stages for psychosis: an individual and transdiagnostic perspective. *Schizophr. Res.* **239**, 95–102 (2022). 34871996 10.1016/j.schres.2021.11.018

[59] Kwapil, T. R. & Barrantes-Vidal, N. Schizotypy: looking back and moving forward. *Schizophr. Bull.* **41**, S366–S373 (2015). 25548387 10.1093/schbul/sbu186 PMC4373633

[60] Schlosser, D. A. et al. Motivational deficits in individuals at-risk for psychosis and across the course of schizophrenia. *Schizophr. Res.* **158**, 52–57 (2014). 25008792 10.1016/j.schres.2014.06.024 PMC4152418

[61] Gandhi, A., Mote, J. & Fulford, D. A transdiagnostic meta-analysis of physical and social Anhedonia in major depressive disorder and schizophrenia spectrum disorders. *Psychiatry Res.* **309**, 114379 (2022). 35123252 10.1016/j.psychres.2021.114379

[62] Wichers, M. The dynamic nature of depression: a new micro-level perspective of mental disorder that meets current challenges. *Psychol. Med.* **44**, 1349–1360 (2014). 23942140 10.1017/S0033291713001979

[63] Wichers, M., Smit, A. C. & Snippe, E. Early warning signals based on momentary affect dynamics can expose nearby transitions in depression: a confirmatory single-subject time-series study. *J. Pers. -Oriented Res.* **6**, 1–15 (2020). 33569148 10.17505/jpor.2020.22042 PMC7842626

[64] Wichers, M., Riese, H., Hodges, T. M., Snippe, E. & Bos, F. M. A narrative review of network studies in depression: what different methodological approaches tell us about depression. *Front. Psychiatry* **12**, 719490 (2021). 10.3389/fpsyt.2021.719490 PMC8581034 34777038

