# Supplementary material for: Diminished anticipatory-consummatory pleasure interplay in high schizotypal traits and subthreshold depression: potential risk for schizophrenia and depression
Source: Schizophrenia (Heidelb). 2026 Apr 4;12(1):48. doi: 10.1038/s41537-026-00746-x (PMC13230509; doi:10.1038/s41537-026-00746-x)
Supplement: Supplementary file 1 — Supplementary Information [file 41537_2026_746_MOESM1_ESM.docx]

**Supplementary Information**

**Supplementary Methods**

**Network accuracy and stability**

We conducted the nonparametric and case-dropping bootstrap analyses with 10,000 bootstraps using the R package ‘bootnet’ (v.1.5.0) to implement the stability estimates of edge weight and node centrality indices stability for the adjusted partial correlation network (Epskamp et al., 2018). Specifically, we estimated the edge weight accuracy with 95% bias-corrected confidence intervals (*CI*s) and the narrower *CI*s indicated a more stable network. Moreover, we estimated the correlation stability (CS) coefficients for the centrality stability. CS-coefficient was defined as the maximum proportion of cases that can be dropped while obtaining a 95% probability that the correlation of the network metrics between the sample with and without dropouts amounts to at least 0.70. It was recommended that the CS-coefficient should be at least 0.25, but preferentially above 0.50 (Epskamp et al., 2018).

In the regularized network for the anticipatory and consummatory affect in the whole sample, we observed relatively narrow bootstrapped *CI*s, indicating the edge weights were accuracy and reliable (Fig.S4). Additionally, the correlation CS-coefficients for the edge weights (CS = .75) and EI centrality (CS = .75) were above .50, suggesting the node centrality of the network was highly stable (Fig.S6).

For the network in the HC individuals, the correlation CS-coefficients for the edge weights (CS = .673) and EI centrality (CS = .673) were above .50. Regarding to the network accuracy and stability for the schizophrenia and depression spectrum, the estimates of edge weight and node centrality indices (the schizophrenia spectrum: CS for the edge weights = .747, for the EI = .672; the depression spectrum: CS for the edge weights = .518, for the EI = .206) were also acceptable and stable.

**Zero-order Correlation Network Sensitivity Analysis**

To examine the robustness of the partial correlation network findings to the choice of association measure, we re-estimated all networks using zero-order Spearman correlations. For each analysis (whole sample, three-group comparison, and spectrum-specific comparisons), covariate-controlled data (sex, age, and reaction times from the three MID conditions) were used to compute zero-order correlation matrices using the cor_auto function in R. Centrality indices (strength, closeness, betweenness, and expected influence) were computed for each zero-order correlation network and compared with those from the corresponding partial correlation (EBICglasso) networks.

**Supplementary Results**

**Demographic characteristics and clinical symptoms in schizophrenia and depression spectrum**

Participants included 115 SCZ patients (48 females) and 83 individuals with schizotypal traits (ST) (52 females) in schizophrenia spectrum (see Materials and Methods for additional information on study participants). As shown in Table 2S, SCZ patients, ST and HC individuals did not match on sex (*χ²* (2) = 9.85, *p* = .007), age (*F* (2, 589) = 160.3, *p* < .0001, η^2^ = .35) and education levels (*F* (2, 588) = 8.48, *p* < .0001, η^2^ = .03). As expected, the SCZ and ST group exhibited higher scores than did HC on the BDI, SPQ (*ps* < .0001, see more details in Table S1).

In depression spectrum, we recruited 60 MDD patients (44 females) and 110 SD individuals (76 females) (see Materials and Methods for additional information on study participants). Similarly, MDD patients, SD and HC individuals did not match on sex (*χ²* (2) = 11.44, *p* = .003), age (*F* (2, 561) = 54.08, *p* < .0001, η^2^ = .16) and education levels (*F* (2, 561) = 24.10, *p* < .0001, η^2^ = .08; see Table3S for demographic information and analyses of group differences in demographics). Additionally, the MDD and SD group exhibited higher scores than did HC on the BDI (*p* < .0001, see more details in Table S2).

Additionally, we compared the demographic characteristic across spectrum, there was significant difference in sex ratio (*χ²* (2) = 16.48, *p* = .0003) and age (*F* (2, 759) = 81.67, *p* < .0001, η^2^ = .18), but not on education levels (*F* (2, 758) = 1.35, *p* = .261, η^2^ = .04).

## Reaction time differences across groups and MID conditions

## *Clinical patients, subclinical individuals, and HC*

To examine whether group differences in consummatory affective ratings could be explained by psychomotor slowing or attentional deficits, we analysed reaction times (RTs) during the consummatory phase of the MID task. A 3 (Group: clinical, subclinical, HC) × 3 (Condition: gain, loss, neutral) mixed-design ANOVA was conducted. Results revealed significant main effects of Group (*F*(2, 778) = 65.10, *p* < .001, η^2^ = .143) and Condition (*F*(2, 1556) = 163.61, *p* < .001, η^2^ = .174), as well as a significant Group × Condition interaction (*F*(4, 1556) = 3.75, *p* = .005, η^2^ = .010). Simple-effect analyses indicated that the clinical group exhibited significantly longer RTs than both the HC and subclinical groups across all three conditions (all *p*s < .001), whereas the subclinical and HC groups did not differ significantly in any condition (all *p*s > .29; Supplementary Table S1). The clinical group showed mean RT increases of approximately 30-39 ms relative to the other two groups, and this slowing was comparable across the gain, loss, and neutral conditions, suggesting a general psychomotor slowing effect rather than a condition-specific deficit.

***Schizophrenia spectrum, Depression spectrum and HC***

A 3 (Group: shizophrenia spectrum, depression spectrum, HC) × 3 (Condition: gain, loss, neutral) mixed-design ANOVA was conducted. Significant main effects were observed for Group (*F*(2, 759) = 28.72, *p* < .001, η^2^ = .070) and Condition (*F*(2, 1518) = 155.74, *p* < .001, η^2^ = .170). However, the Group × Condition interaction was not significant (*F*(4, 1518) = 2.10, *p* = .078, η^2^ = .006), indicating that the pattern of RT differences across groups was consistent across all three MID conditions. The individuals with schizophrenia and depression spectrum exhibited uniformly slower RTs relative to comparison groups, consistent with psychomotor slowing in these populations.

**Zero-order Correlation Network Analysis**

*Whole sample.* The zero-order correlation network for the whole sample (*n* = 821) preserved the core structural features observed in the partial correlation network. Negative associations were evident between reward gain and loss ratings during both the anticipatory and consummatory phases, and a strong positive connection was observed between anticipatory and consummatory reward gain responses. The edge weight profiles of the two networks were highly correlated (Pearson *r* = .841), and of the 30 non-zero edges in the partial correlation network, 93.3% shared the same sign as their zero-order counterparts, with only 2 edges exhibiting sign reversals. Critically, the centrality pattern was fully consistent: anticipatory control affect (Anti_C) and consummatory control affect (Cons_C) exhibited the highest expected influence centrality in the zero-order network, mirroring the partial correlation results (Fig. S2).

*Clinical, subclinical, and HC groups.* The zero-order correlation networks for the three groups showed structural patterns consistent with the corresponding partial correlation networks. The edge weight profiles of the zero-order and partial correlation networks for the Clinical, Subclinical, and HC groups were highly correlated, with Pearson *r* values of 0.794, 0.859, and 0.871, respectively. Of the 24 non-zero edges in the Clinical group, 91.7% shared the same sign as their zero-order counterparts, with only 2 edges exhibiting sign reversals. In the Subclinical group, all 26 non-zero edges (100%) showed consistent signs, while in the HC group, 96.4% of the 28 non-zero edges were consistent with their zero-order counterparts, with just 1 edge exhibiting a sign reversal.The centrality analyses confirmed that consummatory affect under the control condition (Cons_C) retained the highest node strength and expected influence centrality in both the clinical and HC groups, consistent with the partial correlation network findings (Fig. S3).

*Schizophrenia and depression spectra.* The zero-order correlation networks for the SCZ, MDD spectrum and HC groups similarly preserved the key structural features and centrality patterns. The edge weight profiles of the zero-order and partial correlation networks were highly correlated. In terms of Pearson correlation, the SCZ spectrum exhibited a Pearson *r* of 0.824, with 29 non-zero edges, 24 of which (82.8%) showed consistent signs, and 5 edges (17.2%) exhibited sign reversals. The MDD spectrum showed a higher Pearson *r* of 0.889, with 27 non-zero edges, 25 of which (92.6%) demonstrated consistent signs, and 2 edges (7.4%) showed sign reversals. The HC group had a Pearson r of 0.871, with 28 non-zero edges, 27 of which (96.4%) were consistent with their zero-order counterparts, and 1 edge (3.6%) exhibited a sign reversal. Consummatory control affect (Cons_C) maintained the highest node strength and expected influence centrality across the SCZ and MDD spectrum networks (Fig. S4).

**Supplementary Tables**

**Supplementary Table S1.** Post hoc pairwise comparisons of reaction times (ms) across groups (clinical, subclinical, HC) by MID condition.

| **Condition** | **Comparison** | ***Mean A*** | ***Mean B*** | ***Diff*** | ***SE*** | ***t*** | ***p*** |
| --- | --- | --- | --- | --- | --- | --- | --- |
| Gain | Clinical vs. HC | 266.70 | 232.67 | 34.03 | 3.34 | 10.20 | < .001 |
|  | Clinical vs. Subclinical | 266.70 | 232.07 | 34.63 | 3.75 | 9.23 | < .001 |
|  | HC vs. Subclinical | 232.67 | 232.07 | 0.60 | 3.13 | 0.19 | .980 |
| Loss | Clinical vs. HC | 271.69 | 232.89 | 38.80 | 3.50 | 11.09 | < .001 |
|  | Clinical vs. Subclinical | 271.69 | 235.15 | 36.54 | 3.93 | 9.29 | < .001 |
|  | HC vs. Subclinical | 232.89 | 235.15 | -2.26 | 3.28 | -0.69 | .771 |
| Neutral | Clinical vs. HC | 279.68 | 244.24 | 35.44 | 3.61 | 9.81 | < .001 |
|  | Clinical vs. Subclinical | 279.68 | 249.31 | 30.37 | 4.06 | 7.48 | < .001 |
|  | HC vs. Subclinical | 244.24 | 249.31 | -5.07 | 3.39 | -1.50 | .292 |

***Notes.*** Mean A and Mean B denote the means of the first-listed and second-listed groups, respectively, for each comparison; The Diff shows the difference between the means of the two groups, and SE represents the standard error of the mean difference.

**Supplementary Table 2 Demographic and clinical symptoms information in schizophrenia spectrum**

|  | **SCZ**  **(*n* = 115)** | **Schizotypy (*n* = 83)** | **HC**  **(*n* = 394)** | ***χ²/F*** | ***p*** | ***φ/η2*** |
| --- | --- | --- | --- | --- | --- | --- |
| **Sex (male/female)** | 67/48 | 31/52 | 175/219 | 9.85 | .0073 | 0.129 |
| **Age** | 32.32 (8.64) | 21.57 (6.93) | 20.08 (5.59) | 160.3 | < .0001 | 0.35 |
| **Education (years)** | 12.12 (2.64) | 13.77 (1.53) | 12.84 (2.99) | 8.48 | < .0001 | 0.03 |
| **BDI** | 10.55 (10.24) | 7.80 (4.82) | 5.99 (4.58) | 16.28 | < .0001 | 0.07 |
| **SPQ** | 32.20 (29.09) | 41.36 (9.08) | 19.63 (9.36) | 153.4 | < .0001 | 0.49 |
| **Dosage of drug (Chlorpromazine equivalence, mg/d)** | 289.42 (206.75) |  |  |  |  |  |

***Notes.*** BDI Beck Depression Inventory, SPQ Schizotypal Personality Questionnaire, SCZ schizophrenia

**Supplementary Table 3 Demographic and clinical symptoms information in depression spectrum**

|  | **MDD**  **(*n* = 60)** | **SD**  **(*n* = 110)** | **HC**  **(*n* = 394)** | ***χ²/F*** | ***p*** | ***φ/η2*** |
| --- | --- | --- | --- | --- | --- | --- |
| **Sex (male/female)** | 16/44 | 34/76 | 175/219 | 11.44 | .003 | 0.142 |
| **Age** | 26.98 (7.64) | 18.06 (3.15) | 20.08 (5.59) | 54.08 | < .0001 | 0.16 |
| **Education (years)** | 15.31 (2.56) | 12.11 (2.95) | 12.84 (2.99) | 24.10 | < .0001 | 0.08 |
| **BDI** | 25.23 (11.01) | 22.69 (7.03) | 5.99 (4.58) | 433.50 | < .0001 | 0.64 |
| **SPQ** |  | 25.25 (4.99) | 19.63 (9.36) | 1.43 | .232 | 0.006 |
| **DOI (month)** | 5.88 (10.62) |  |  |  |  |  |
| **Dosage of drug (Fluoxetine equivalence, mg/d)** | 10.10 (16.25) |  |  |  |  |  |

***Notes.*** BDI Beck Depression Inventory, SPQ Schizotypal Personality Questionnaire, MDD major depressive disorder

**Supplementary Figures**


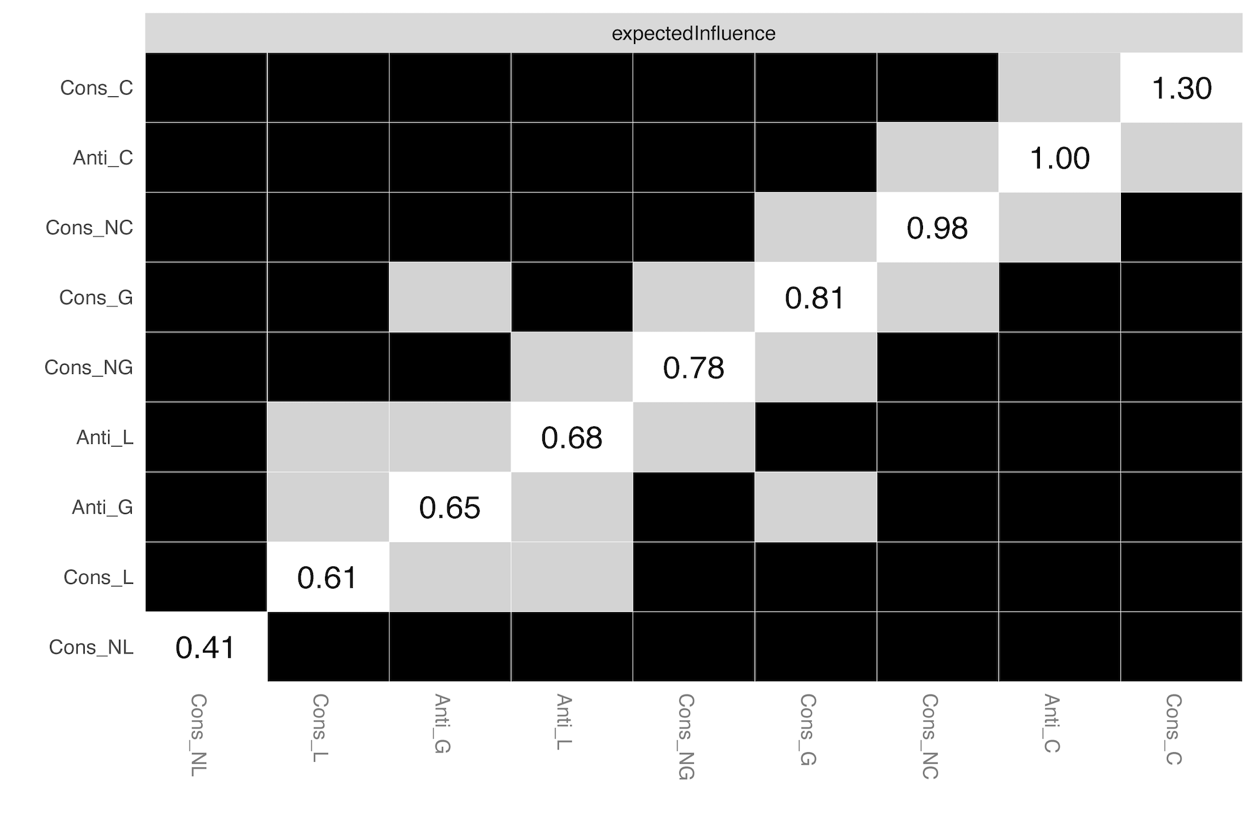


**Fig.S1** Non-parametric bootstrapped difference test for expected influence in the network for the whole sample. Gray boxes indicate no difference between nodes, while black boxes indicate significant difference (α = .05).

**Fig.S2** Centrality plots depicting the standardized node strength, closeness, betweenness and expected influence for the network in the whole sample, (A) Partial Correlation Networks (EBICglasso) and (B) Zero-order Correlation Networks with sex, age and RTs as covariates.

**Fig.S3** Centrality plots depicting the standardized node strength, closeness, betweenness and expected influence for the network in the clinical, subclinical and HC samples, (A) Partial Correlation Networks (EBICglasso) and (B) Zero-order Correlation Networks with sex, age and RTs as covariates.

**Fig.S4** Centrality plots depicting the standardized node strength, closeness, betweenness and expected influence for the network in the schizophrenia, depression spectrum and HC samples, (A) Partial Correlation Networks (EBICglasso) and (B) Zero-order Correlation Networks with sex, age and RTs as covariates.


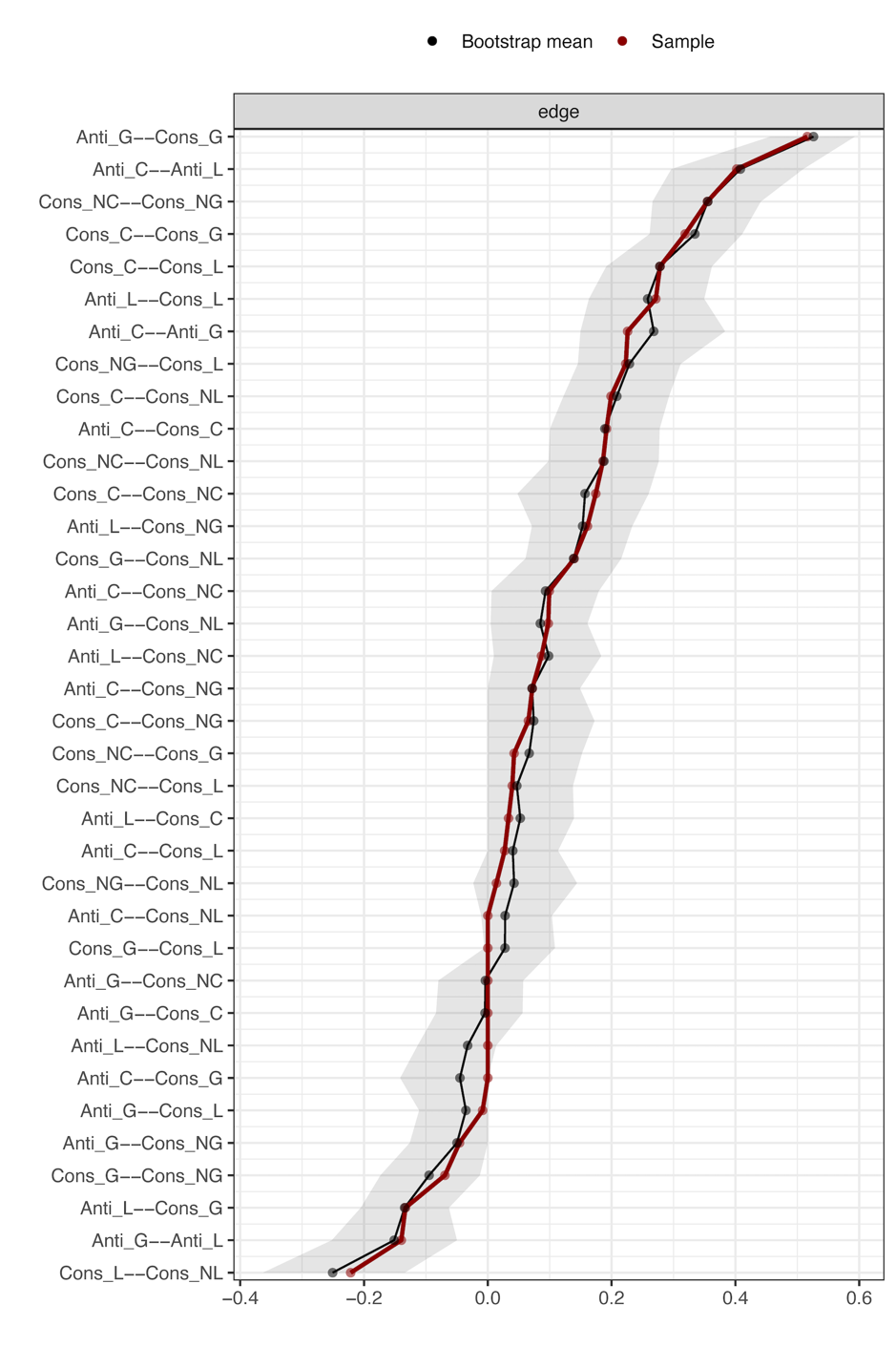


**Fig.S5** Non-parametric bootstrapped confidence intervals of estimated edges for the anticipatory and consummatory affect. The red line represents the edge and the gray area indicates that 95% bootstrapped confidence interval.


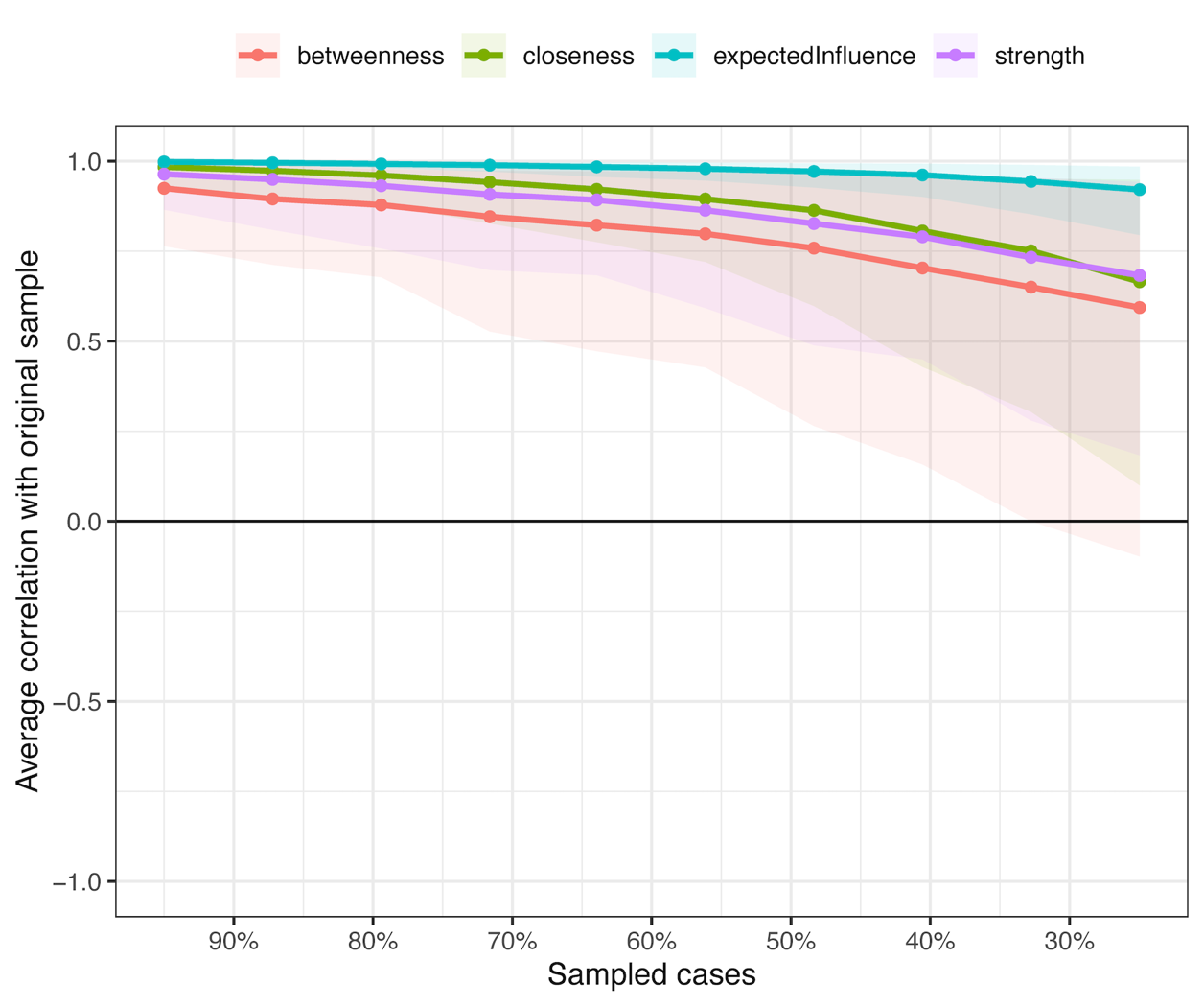


**Fig.S6** Stability of centrality indexes by case dropping subset bootstrap in the network for the whole sample.

**Reference**

Epskamp, S., Borsboom, D., & Fried, E. I. (2018). Estimating psychological networks and their accuracy: A tutorial paper. *Behavior Research Methods*, *50*(1), 195–212. https://doi.org/10.3758/s13428-017-0862-1
